# Supplementary material for: Exploiting the Affimer platform against influenza A virus
Source: mBio. 2024 Jul 22;15(8):e01804-24. doi: 10.1128/mbio.01804-24 (PMC11323568; doi:10.1128/mbio.01804-24)
Supplement: Supplemental Material — Fig. S1 to S8 and Tables S1 and S2. [file mbio.01804-24-s0001.pdf]

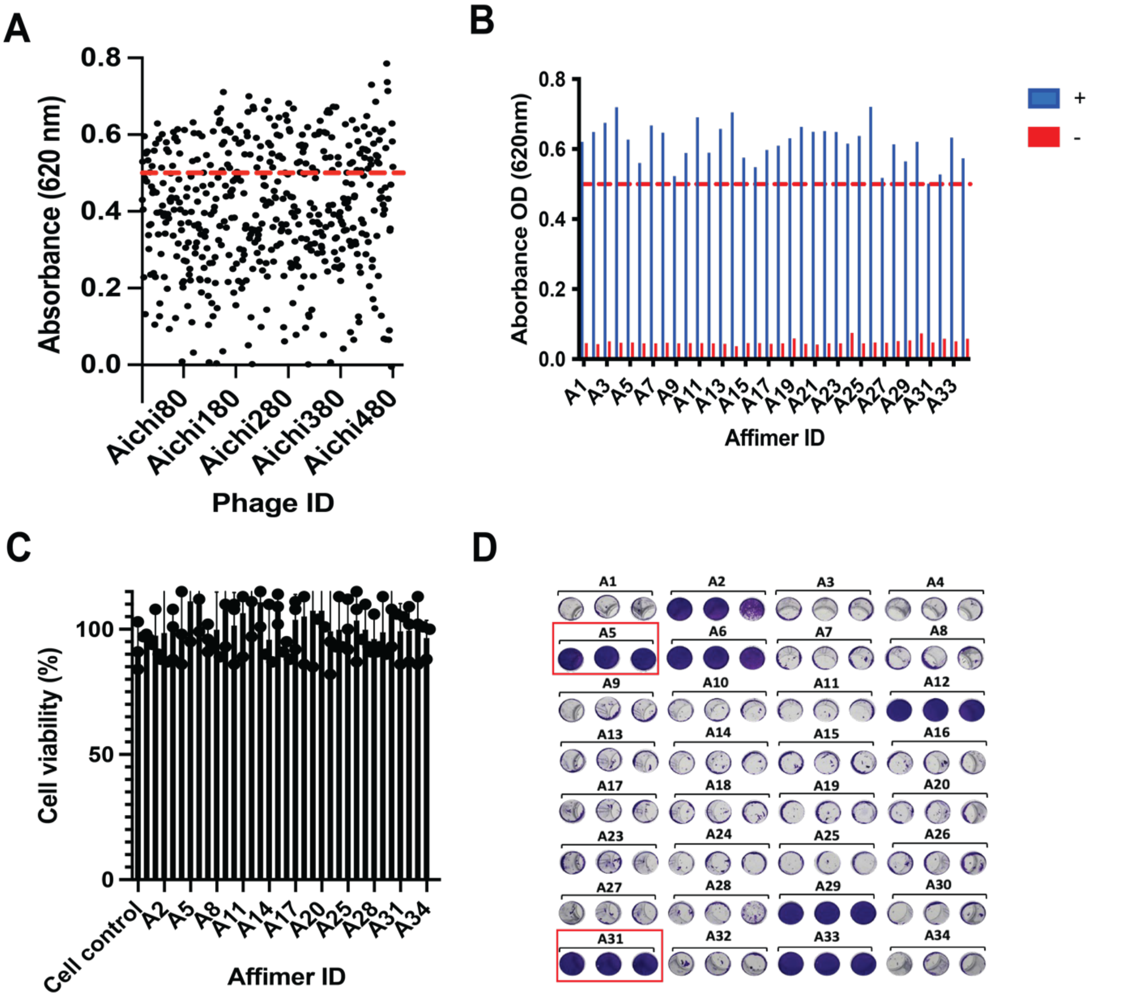

Supplementary Figure 1. Isolation of Affimer molecules

(A) Phage-ELISA results showing isolated phage absorbance profiles to immobilised monomeric A/Aichi/1968 (H3N2*).* Red dashed line indicates the cut-off of 0.5 for 192 phage candidates to be sequenced (absorbance at 620 nm) **(B)** ELISA absorbance profiles of the 34 unique Affimer molecules, post-sequencing resulting from the 192 phage candidates selected. Blue bars represent absorbance of the selected phages; red bars are absorbances of the negative controls. **(C)** Cell viability in MDCK cells following treatment with 100 µM of isolated Affimers for 72 hrs. **(D)** Neutralisation assay of isolated Affimer molecules. A/Aichi/1968 (H3N2) virus was pre-incubated with 3.7 µM of each Affimer (50 µg/ mL) for 72 hrs before fixing and staining MDCK cell monolayers.

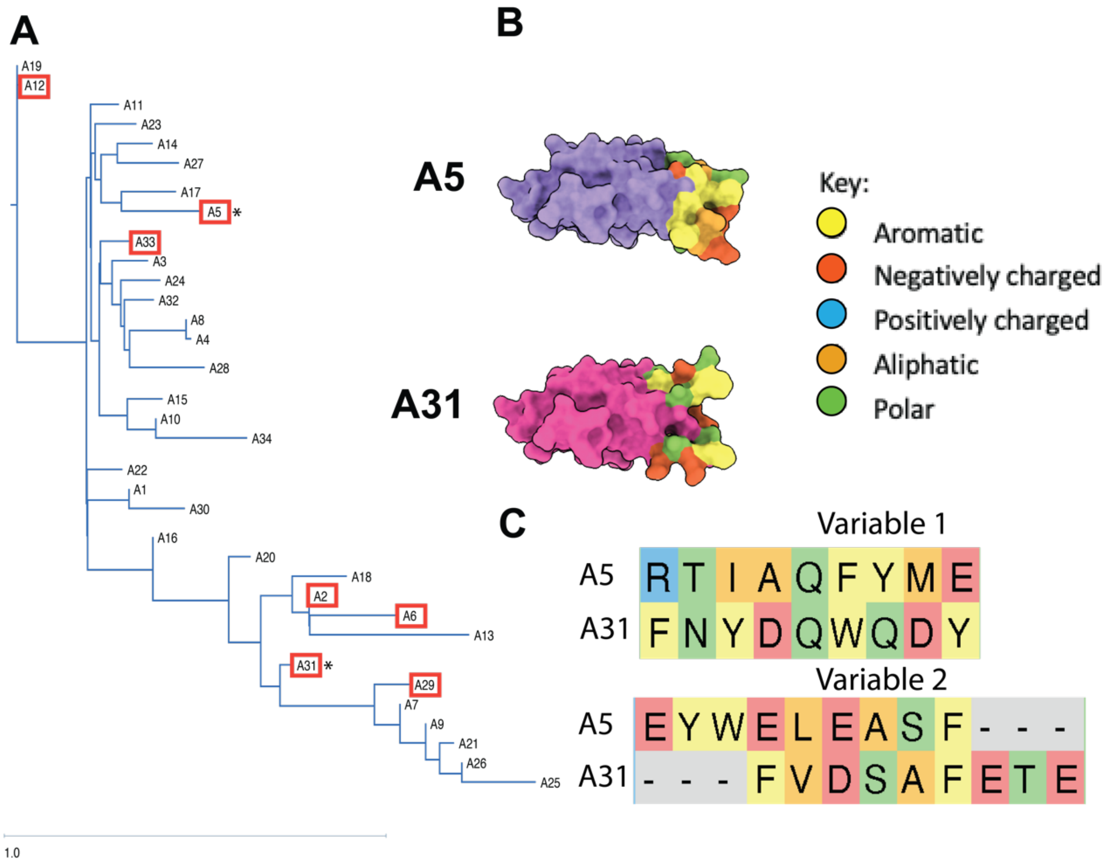

Supplementary Figure 2. Initial characterisation of isolated Affimer molecules

(A) Phylogenetic tree of the variable region from isolated Affimers made using multiple sequence alignment (ClustalOmega). Neutralising Affimers (at 3.7 μM; 50 μg/mL) are encompassed by a red box. **(B)** AlphaFold2 surface representation structures of Affimers A5 and A31 (blue and purple, respectively), with amino acids of the variable regions coloured based on the properties of the side-chain. **(C)** Alignment of variable regions of A5 and A31, coloured based on side-chain properties.

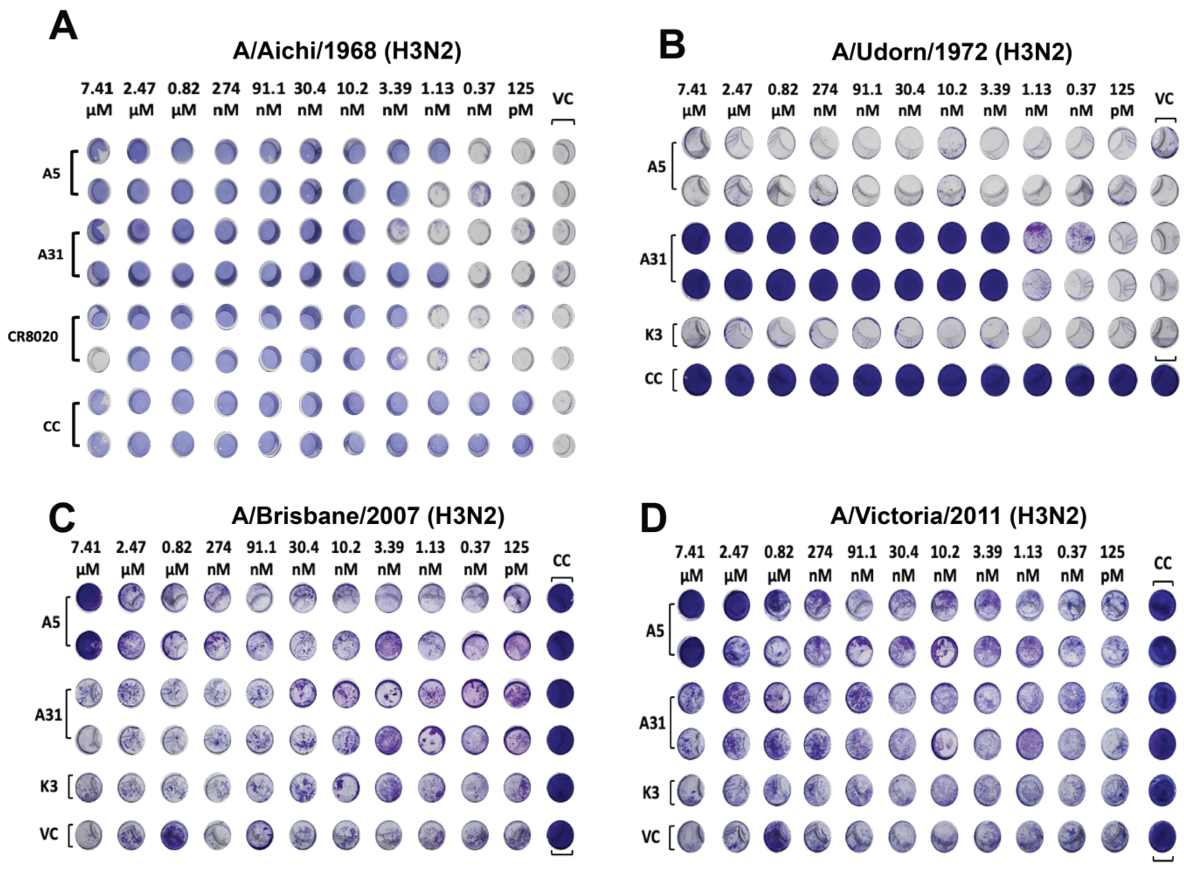

Supplementary Figure 3. $TCID_{50}$ neutralisation of H3N2 viruses

$TCID_{50}$ assay against IAV A/Aichi/1968 (H3N2) **(A)**, A/Udorn/1972 (H3N2) **(B)**, *A/Brisbane/2007 (H3N2)* **(C)**, and *A/Victoria/2011 (H3N2)* **(D),** for A5, A31, mAb control CR8020, control Affimer K3, cell only control (CC) and virus control (VC)

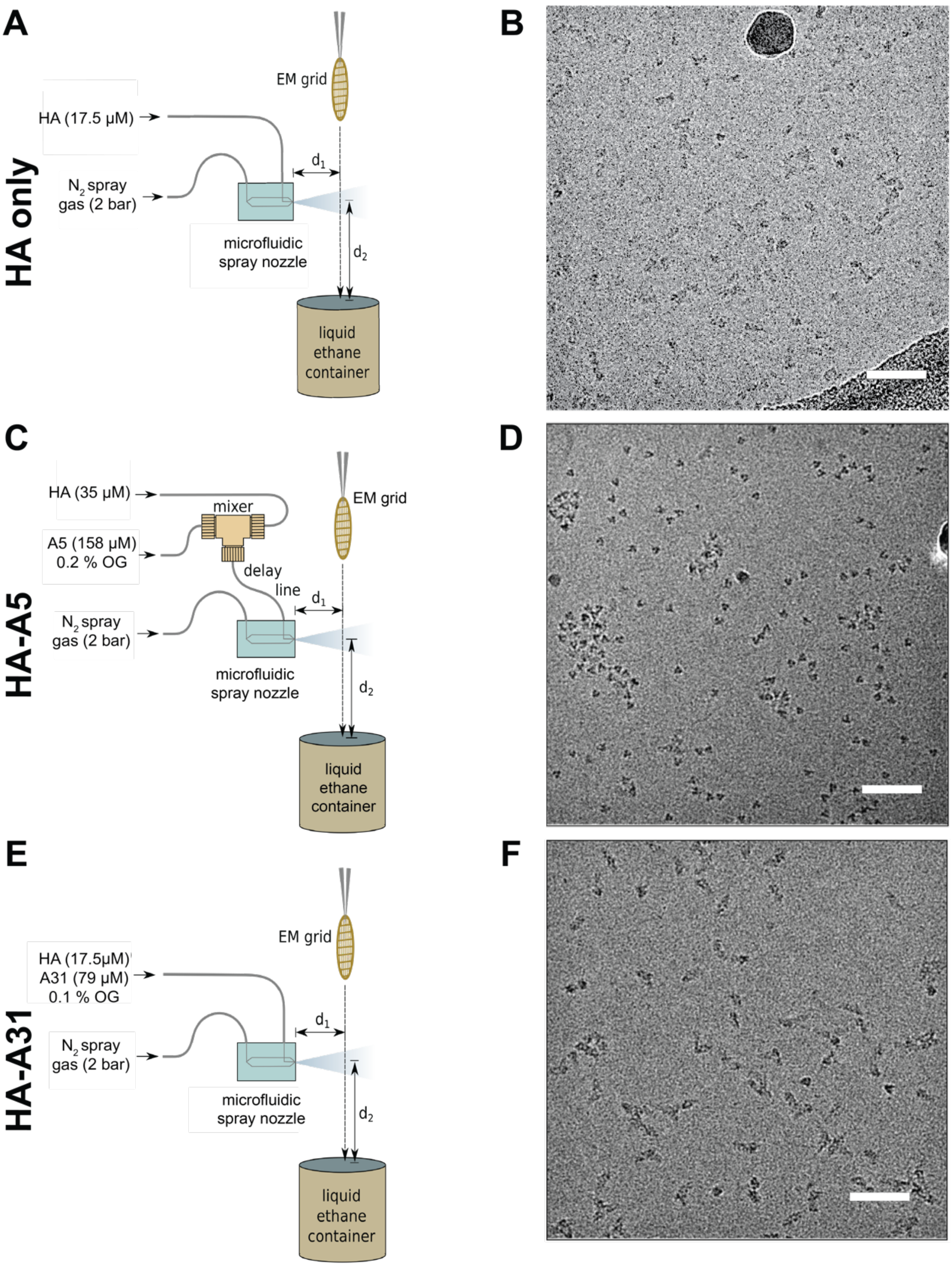

Supplementary Figure 4. Cryo-electron microscopy sample preparation

Cryo-EM grid preparation set-up for HA only, H3-A5 complex, and H3-A31 complex, using custom built rapid-spray and vitrification set-up. **(A)** HA only spray and vitrification set-up. **(B)** Representative micrograph for HA-only. **(C)** H3-A5 complex spray and vitrification set-up. **(D)** Representative micrograph for H3-A5 complex. **(E)** H3-A5 complex spray and vitrification set-up. **(F)** Representative micrograph for H3-A31 complex. OG is octyl glucoside, d_1 is the nozzle-grid distance, d_2 is the nozzle-ethane distance.

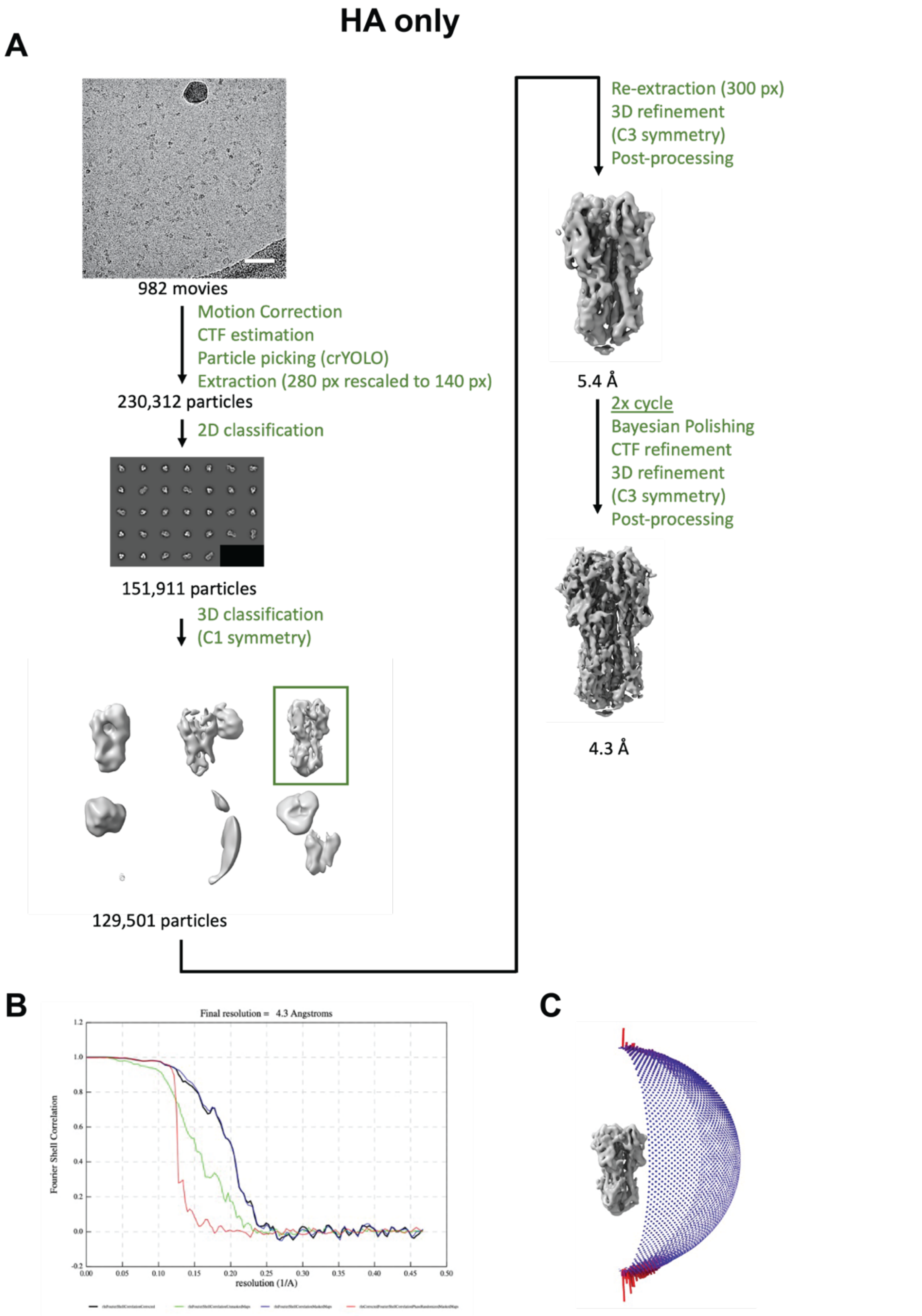

Supplementary Figure 5. Processing of H3 only cryo-EM data
Processing pipeline of HA only cryo-EM data. All processing jobs were carried out using RELION3.1, resulting in a final global resolution of 4.3 Å. **(A)** Representative micrograph of HA cryo-EM grid acquisition and image processing pipeline. **(B)** Gold-standard Fourier shell correlation (GFSC) curve generated in RELION. **(C)** Representation of particle orientation distribution. Red represents views with many particles, while blue represents views with few particles.

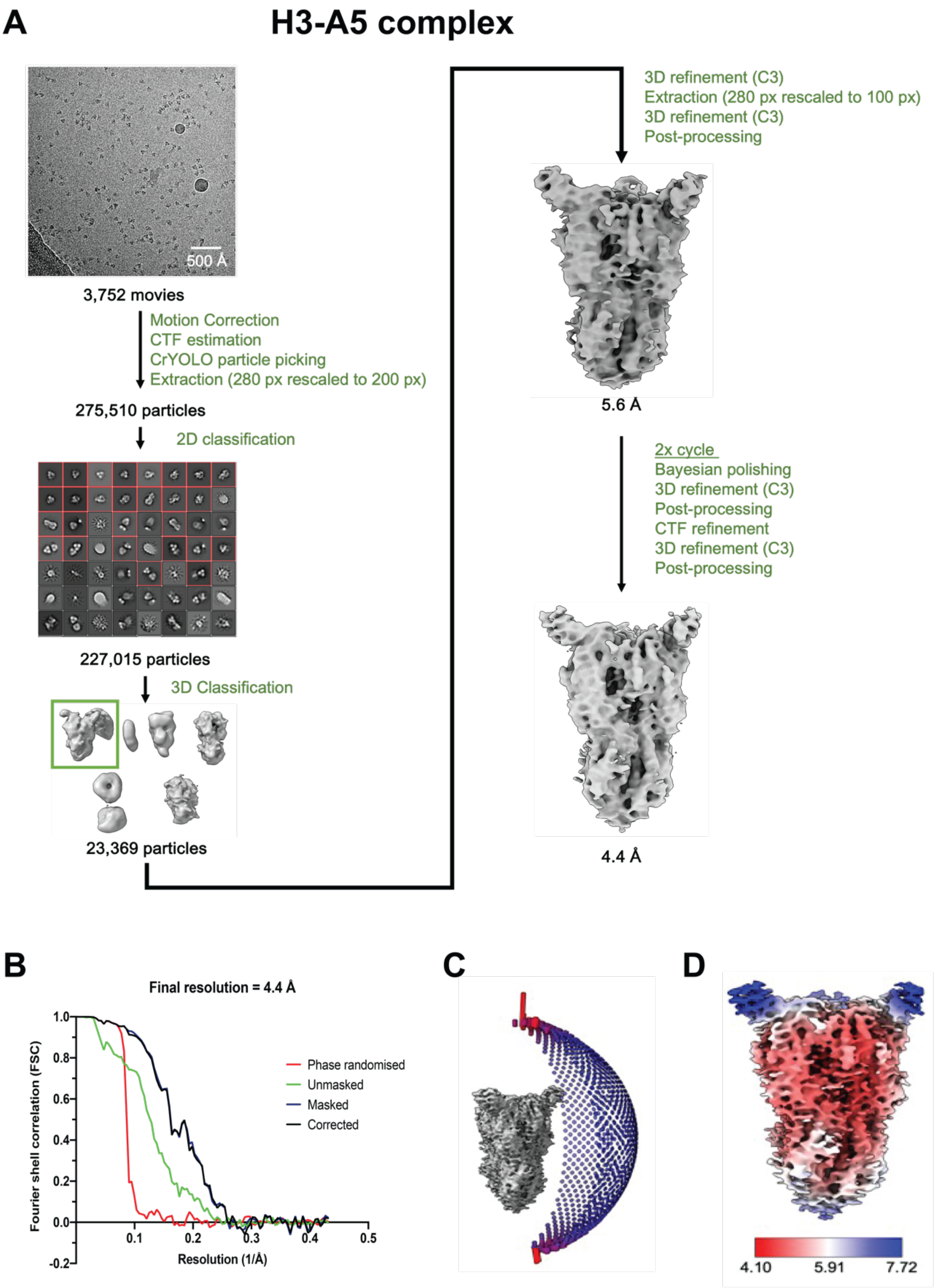

Supplementary Figure 6. Processing of H3-A5 cryo-EM data

Processing pipeline of H3-A5 complex cryo-EM data. All jobs were carried out using RELION3.1, resulting in a final global resolution of 4.4 Å **(A)** Representative micrograph of H3-A5 complex cryo-EM grid acquisition and image processing pipeline. Scale bar represents 50 nm. **(B)** GFSC curve generated in RELION. **(C)** Representation of particle orientation distribution. Red represents views with many particles, while blue represents views with few particles. **(D)** Local resolution of the H3-A5 complex.

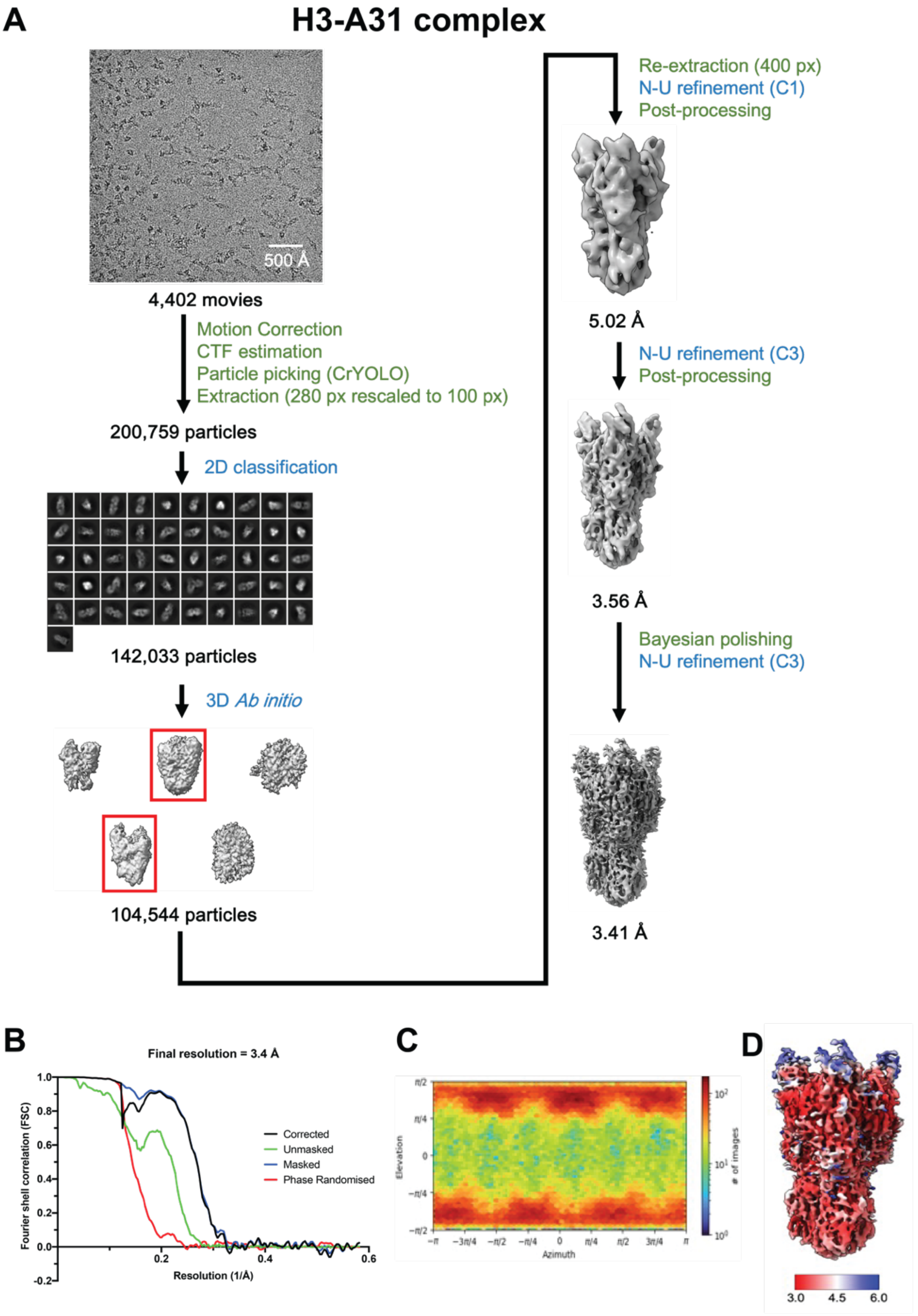

Supplementary Figure 7. Processing of H3-A31 cryo-EM data
Processing pipeline of H3-A31 complex cryo-EM data. Processing jobs were carried out in both RELION3.1 and cryoSPARC (job description in green font or blue font, respectively), resulting in a final global resolution of 3.4 Å. **(A)** Representative micrograph of H3-A31 complex cryo-EM grid acquisition and image processing pipeline. **(B)** GS-FSC curve generated in RELION. **(C)** Representation of particle orientation distribution. Red represents views with many particles, while green represents views with few particles. **(D)** Local resolution of the H3-A31 complex.

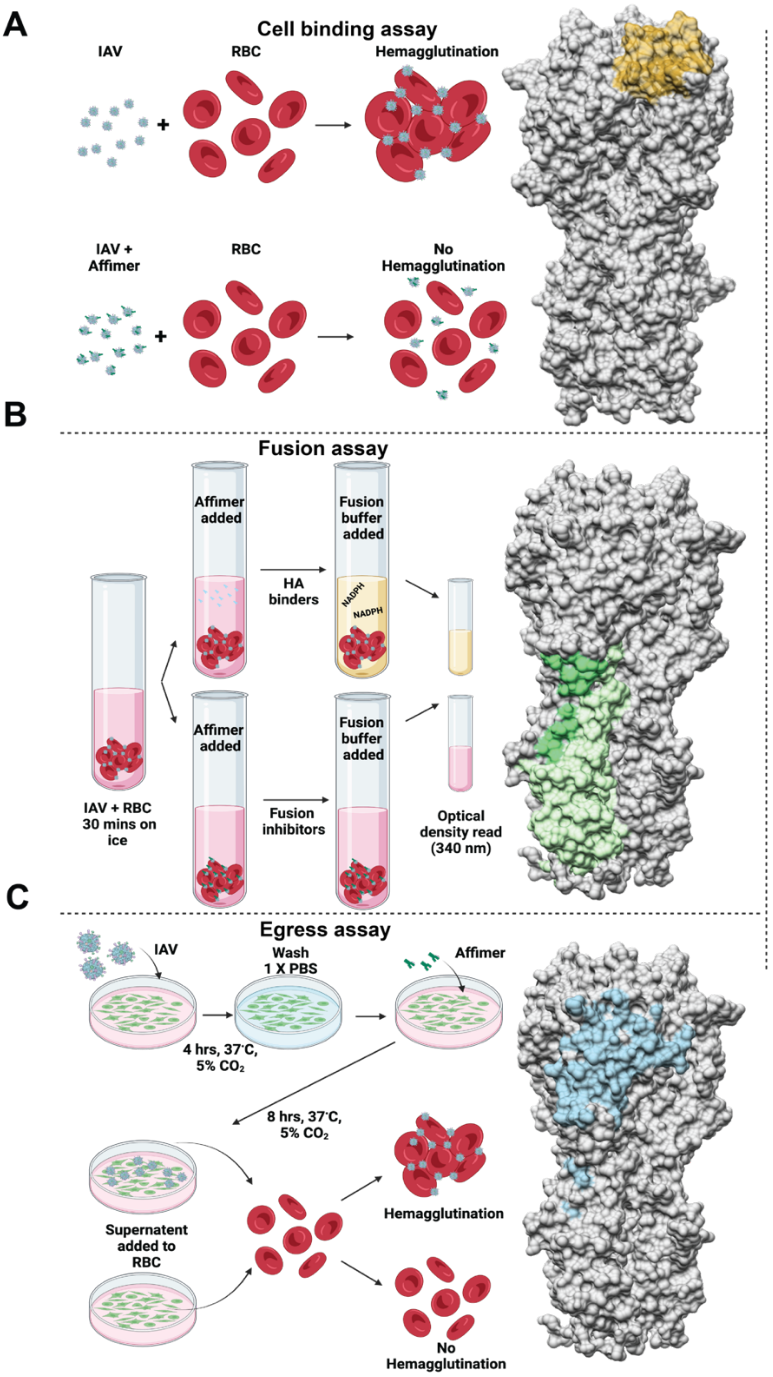

Supplementary Figure 8. Schematic representation of mechanism of inhibition assays for characterisation of Affimer neutralisation

Schematic representations of **(A)** a hemagglutination assay to determine inhibition via interruption of IAV cell binding; **(B)** a fusion assay to determine inhibition via interruption of IAV viral-host cell membrane fusion; and **(C)** an egress assay to determine inhibition via interruption of IAV egress from infected host cells.

	HA only	H3-A5 complex	H3-A31 complex
N2 spray gas pressure	2 bar	2 bar	2 bar
Delay-line	-	20 mm tubing, 381 µm diameter	-
Sample flow rate	5.2 µl/s	4.2 µl/s	4.2 µl/s
Plunge speed	1.9 m/s	1.5 m/s	1.5 m/s
Nozzle-grid distance (d1)	10 mm	12 mm	12 mm
Nozzle-ethane distance (d2)	22 mm	22 mm	22 mm

Supplementary Table 1. Experimental settings for preparing cryo-EM grids of HA only, H3-A5 complex, and H3-A31 complex

	H3 HA only	H3-A5	H3-A31
**Data Collection:**			
Microscope	FEI Titan Krios	FEI Titan Krios	FEI Titan Krios
Voltage (keV)	300	300	300
Detector	Gatan K2	FEI Falcon IV	FEI Falcon IV
Magnification	x 130k	x 96k	x 96k
Defocus range	-3 to -5	-2 to -4	-2 to -4
Pixel size (Å)	1.07	0.83	0.83
Electron dose (e^-/Å^2)	61.2	56.2	56.2
Electron dose per frame (e^-/Å^2)	1.9	1.3	1.3
Exposure (sec)	7	5.99	5.99
No. of frames	32	43	43
No. of micrographs	982	3,752	4,402
**Data processing:**			
Symmetry point group	C3	C3	C3
Final particle number	129,501	206,111	104,544
Map average resolution (Å, 0.143 FSC threshold)	4.3	4.4	3.4
Map sharpening B-factor (Å^2)	-200	-100	-80
**Refinement:**			
Initial model (PDB code)	-	-	4FNK
**Model composition:**			
Non-hydrogen atoms	-	-	14,277
Amino acid residues	-	-	1,779
**R.M.S.D. from ideal geometry:**			
Bond lengths (Å)	-	-	0.005
Bond angles (º)	-	-	0.888
**Validation:**			
Clashscore	-	-	9.03
Rotamer outliers (%)	-	-	0.00
**Ramachandran plot statistics:**			
Favored (%)	-	-	92.12
Allowed (%)	-	-	7.88
Outliers (%)	-	-	0.0
**FSC model-map (Å, 0.5 threshold)**	-	-	3.92

Supplementary Table 2. Summary of data acquisition, processing and statistics of final atomic models generated from cryo-EM data.
